# A62 PROTEASE-ACTIVATED RECEPTOR-2 ACTIVATION ENHANCES EPITHELIAL WOUND HEALING THROUGH SRC PATHWAY-DEPENDENT CELL MIGRATION

**DOI:** 10.1093/jcag/gwab049.061

**Published:** 2022-02-21

**Authors:** L Lucena Périco, W MacNaughton

**Affiliations:** Physiology and Pharmacology, University of Calgary Cumming School of Medicine, Calgary, AB, Canada

## Abstract

**Background:**

Protease-activated receptors (PARs) and their activating enzymes play a role in inflammatory bowel disease (IBD) pathogenesis, but the specific roles of PAR2 in disease initiation and progression remain unclear. Interestingly, PAR2 activation has both pro-proliferative and pro-migratory effects and could be involved with restoration of the epithelial barrier following injury. We previously showed that the PAR2 activation increase the epithelial wound healing through ERK, PI3K and JNK pathways. However, the role of Src kinase, which is also activated by PAR2, is not known.

**Aims:**

We hypothesized that PAR-2 activation induces a wound healing response in intestinal epithelial cells through Src activity.

**Methods:**

Circular wounds were made in T84 colonic epithelial cells monolayers. Wounded monolayers were treated with the PAR2 activating peptide, 2-furoyl-LIGRLO (2fLI, 5 μM), or the inactive control reverse-sequence peptide, 2-furoyl-OLRGIL (2fO, 5 μM), and live cell imaging was used to record wound closure over a 12 or 24-hr period. The specificity of PAR2 was assessed with a PAR2 inhibitor (GB88). Proliferation and cytotoxicity were measured using EdU and TUNEL assays. The mechanism of action was evaluated using inhibitors of FAK (PF57328 and FAK14) and Src (PP2) and western blot (WB) was used to confirm the protein levels [p-FAK (Y397; Y576/577) and p-Src (Y416)]. For immunofluorescence, images of E-cadherin and F-actin or p-FAK were taken to capture the entire wound border and surrounding cells.

**Results:**

PAR2 activation by 2fLI promoted wound closure compared to 2fO or vehicle control at the 12 and 24 hr timepoints ( *p*<0.05). PAR2’s ability to enhance wound closure was blocked with the PAR2 inhibitor, GB88 ( *p*<0.01). PAR2 activation had no effect on proliferation at the wound-edge and did not cause apoptosis, but enhanced lamellipodia/filopodia formation ( *p*<0.001), indicating that PAR2 might program the cells toward a migratory phenotype rather than a proliferative phenotype. When we investigated the mechanisms of action, PAR2 activation did not induce focal adhesion kinase (FAK) expression in T84 cells and inhibition of FAK by selective inhibitors (PF573288 and FAK14) did not alter PAR2-induced wound healing. The immunofluorescence for p-FAK (Y397) and WB (Y937 and Y576/577) confirmed these results. However, the Src tyrosine kinase inhibitor (PP2, *p*<0.0001) inhibited PAR2-induced wound healing. PAR2 activation increased Src phosphorylation (Y416, *p*<0.05).

**Conclusions:**

PAR2 activation drives wound healing in part via Src tyrosine kinase activity, but independently of FAK activity. These findings provide a further mechanism whereby PAR2 can participate in the resolution of intestinal wounds in gastrointestinal inflammatory diseases.

**Funding Agencies:**

CAG, CIHR

